# Spatio-temporal Index Based on Time Series of Leaf Area Index for Identifying Heavy Metal Stress in Rice under Complex Stressors

**DOI:** 10.3390/ijerph17072265

**Published:** 2020-03-27

**Authors:** Yibo Tang, Meiling Liu, Xiangnan Liu, Ling Wu, Bingyu Zhao, Chuanyu Wu

**Affiliations:** 1 School of Information Engineering, China University of Geosciences, Beijing 100083, China; delicktang@cugb.edu.cn (Y.T.); liuxn@cugb.edu.cn (X.L.); wuling@cugb.edu.cn (L.W.); wuchy@cugb.edu.cn (C.W.); 2 Faculty of Geographical Science, Beijing Normal University, Beijing 100875, China; zby3135@sina.com

**Keywords:** heavy metal stress, rice growth, dynamic time warping, Moran’s I

## Abstract

Crops under various types of stresses, such as stress caused by heavy metals, drought and pest/disease exhibit similar changes in physiological-biochemical parameters (e.g., leaf area index [LAI] and chlorophyll). Thus, differentiating between heavy metal stress and nonheavy metal stress presents a great challenge. However, different stressors in crops do cause variations in spatiotemporal characteristics. This study aims to develop a spatiotemporal index based on LAI time series to identify heavy metal stress under complex stressors on a regional scale. The experimental area is located in Zhuzhou City, Hunan Province. The situ measured data and Sentinel-2A images from 2017 and 2018 were collected. First, a series of LAI in rice growth stages was simulated based on the WOrld FOod STudies (WOFOST) model incorporated with Sentinel 2 images. Second, the local Moran’s I and dynamic time warping (DTW) of LAI were calculated. Third, a stress index based on spatial and temporal features (SIST) was established to assess heavy metal stress levels according to the spatial autocorrelation and temporal dissimilarity of LAI. Results revealed the following: (1) The DTW of LAI is a good indicator for distinguishing stress levels. Specifically, rice subjected to high stress levels exhibits high DTW values. (2) Rice under heavy metal stress is well correlated with high-high SIST clusters. (3) Rice plants subjected to high pollution are observed in the northwest of the study regions and rice under low heavy metal stress is found in the south. The results suggest that SIST based on a sensitive indicator of rice biochemical impairment can be used to accurately detect regional heavy metal stress in rice. Combining spatial-temporal features and spectral information appears to be a highly promising method for discriminating heavy metal stress from complex stressors.

## 1. Introduction

Heavy metals, such as Cadmium (Cd), in paddy rice fields can efficiently accumulate in rice grain, straws, and roots [1] due to their high ingestion rate [2], disturb various physiological processes [3], and ultimately have adverse effects on human health [4]. Although field surveys can accurately detect heavy metal concentrations in paddy fields [5], they are often time consuming and expensive and do not facilitate mapping the extent of heavy metal contamination for lager regions. Remote sensing techniques enable the examination of the influence of heavy metal contamination on rice at a large scale.

Researchers have attempted to measure heavy metal stress levels by using physiological and spectral features, because heavy metal contaminants have direct or indirect influences on physiological parameters such as leaf area, dry weight, photosynthetic efficiency, and transpiration rate; these influences, in turn affect several spectral values in remote sensing images. Indices based on hyperspectral [6] or multispectral [7] images reflecting stress levels in rice, canopy–air temperature difference [8], crop growth models, like the WOrld FOod STudies (WOFOST) model [9], and the components of time series decomposition [10] have been proposed to discriminate heavy metal stress in rice on the basis of remote sensing images.

Clues to heavy metal stress in remote sensing images are usually difficult to find because the spectral characteristics of these signals are similar to those of other types of stresses, such as pests and disease [11,12]. Some studies solve this problem by considering temporal information on the basis of multi-temporal images or time series decomposition at field levels [13]. Although some researchers have used temporal information to identify heavy metal stress, few have analyzed the year-to-year dissimilarity and spatial patterns of heavy metal stress in rice. Dynamic time warping (DTW), a time series dissimilarity measurement that emphasizes the difference in amplitude and eliminates temporal scaling and shifting effects, is introduced in this study to solve the problem of comparing series with the different timings of rice phenological stages (e.g., seeding and transplanting) [14] in adjacent years when measuring the year-to-year dissimilarity between rice leaf area index (LAI) series. Moran’s I, a local index of spatial autocorrelation [15], is then calculated on the basis of the DTW results to find spatial patterns in the year-to-year change of the rice LAI series.

DTW, which was first introduced in 1960s [16], is a measurement of time series dissimilarity and can be applied to speech recognition, gesture recognition, signature matching, and music and signal processing [17]. DTW finds the optimal warping path between two time series by minimizing the effects of shifting and distortion in time and calculates the distance between them. DTW is used in remote sensing to classify land cover types with temporal information [18,19,20,21].

Given that frequent cloud coverage in the study area contaminates pixels in satellite images, the available remote sensing images cannot meet the demand of time series analysis. The WOFOST crop growth model is thus used in this study to generate a dense time series because this model can simulate annual crop growth with a 1-day interval [22], and incorporate with remote sensing images [23,24,25]. The model takes crop physiological parameters, as well as meteorological conditions, crop species, and management factors, into consideration. The output of the WOFOST model includes the time series of LAI and dry weights of roots, stems, leaves, and storage organs [26,27]. The time series generated by this model more closely resembles actual rice growth than that generated by mathematical interpolation methods.

In this work, DTW is chosen as the dissimilarity measurement between WOFOST-simulated LAI series of rice pixels. We then analyze the local spatial autocorrelation of DTW dissimilarity with Anselin Local Moran’s I [28], and find that rice pixels under heavy metal stress tend to be clustered. Finally, SIST, a heavy metal stress index that considers stress levels measured by DTW, temporal dissimilarity, and local Moran’s I of DTW is proposed to examine the severity of heavy metal stress.

## 2. Study Area and Data

### 2.1. Study Area

**Figure 1:**
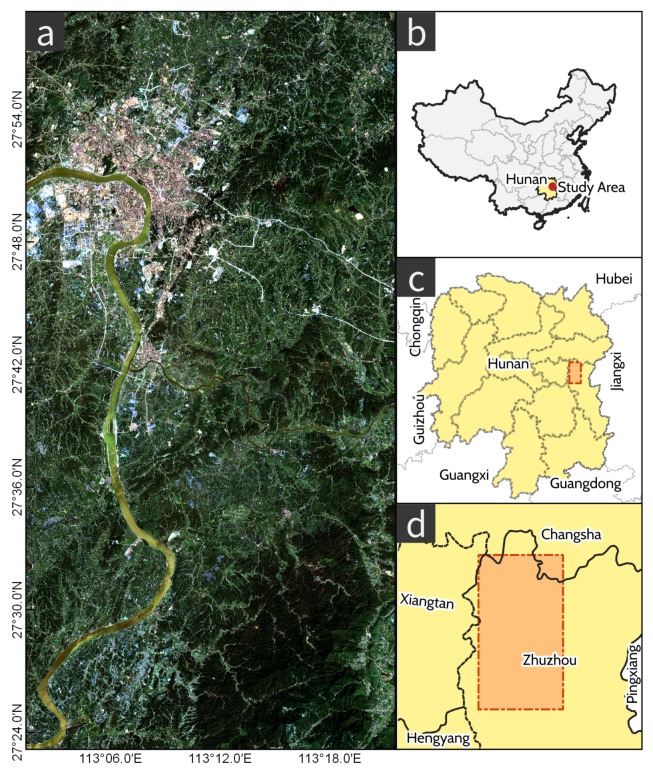
Map of the study area (**a**): Raw Sentinel 2 scene on Sep 15, 2017. (**b**): Study area in relation to China. (**c**): Study area in relation to Hunan Province, China. (**d**): Study area and surrounding cities.

The study area (113°2${}^{\prime }$25${}^{\prime \prime }$ E, 27°25${}^{\prime }$50${}^{\prime \prime }$ N–113°21${}^{\prime }$56${}^{\prime \prime }$ E, 27°56${}^{\prime }$52${}^{\prime \prime }$ N) shown in Figure 1 is located in Zhuzhou, the second largest city in Hunan province, central China. The area belongs to a humid subtropical climate zone with an annual average temperature of 17.9 °C and precipitation of 1504 mm, which is suitable for rice growing. Xiangjiang River, one of the largest tributaries of China’s Yangtze River, which flows through Zhuzhou, is the major source of agriculture water. The city is a primary grain production area in China. Zhuzhou is also an industrial city that specializes in metallurgy, machine manufacturing, chemicals and building materials, all of which can discharge heavy metal contaminants to the surrounding water, soil, and atmosphere. Rice can absorb heavy metals via root uptake and atmospheric decomposition.

### 2.2. Data Collection

#### 2.2.1. Point Data

Meteorological data and crop growth parameters are used to localize the WOFOST models. The growth of rice can be simulated by taking in-situ data as WOFOST inputs.

Meteorological data during the growing season (June 1 to September 30) from 2017 to 2018, including daily maximum temperature ($T_{\max}$), daily minimum temperature ($T_{\min}$) and daily sunshine hours, were acquired from Zhuzhou Meteorological Station and can be accessed via the National Meteorological Information Center (http://data.cma.cn/en).

LAIs were collected between July 2017 and August 2017 using a LAI-2000 plant canopy analyzer. LAI in each sample point was measured thrice and the mean value of the measurements was taken as the final LAI of a sample and used to incorporate the LAI and remote sensing images. We first modelled the relationship between measured LAIs and satellite-derived NDVIs and calculated images of LAI according to the model. Satellite-derived LAIs were then taken as the crop growth parameter inputs of the WOFOST model.

We used soil heavy metal concentration and factory coordinates for accuracy measurements in this study. Soil heavy metal concentrations were collected at the field level for validation. The concentrations of Cd in soil were determined by using inductively coupled plasma mass spectrometry (7500a, Agilent Technologies, USA). During analysis process, the accuracy of the samples were controlled by a program blank and soil composition analysis standard material GSS-13 (National Standards Research Center). Each soil sample was measured for thrice, and the concentration is presented as the mean value of these measurements.

The coordinates of factories in Zhuzhou were collected from AutoNavi, one of the largest online map service providers in mainland China. This provider includes millions of up-to-date points of interest data. GCJ-02, the coordinate system of AutoNavi, was transformed into WGS-84 using an open source Python version of eviltransform [29] to maintain the same spatial reference used by Sentinel 2 images. These coordinates were used for validation.

#### 2.2.2. Satellite Imagery

In this study, we used Sentinel 2 images taken during the growing season (June 1 to Sep 30) from 2017 to 2018. Compared with Landsat, Sentinel 2 has higher revisit frequency (10 days for a single satellite and 5 days for combined constellation) and a spatial resolution of up to 10m. Sentinel 2 images were acquired from Google Earth Engine’s Javascript API [30].

**Figure 2:**
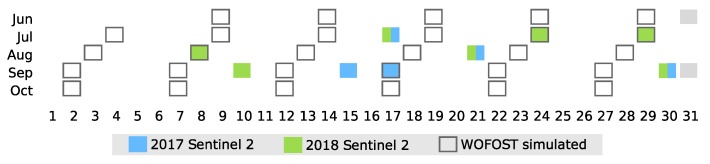
Acquisition dates of satellite images and dates of leaf area index (LAI) images simulated with the WOrld FOod STudies (WOFOST) model.

To generate a LAI time series with increased accuracy, we filtered all Sentinel 2 scenes with a cloud coverage less than 10% in the study area and removed clouds with the cloud mask band. The acquisition dates of Sentinel 2 images and dates of WOFOST simulated LAIs are illustrated in Figure 2. Then, we calculated NDVIs from the cloud-free image collection and transformed the values into LAI.

## 3. Methods

Previous studies have shown that heavy metals are stable across space and time [31], whereas other stresses, such as pests and diseases, show more variability. Therefore, discriminating heavy metal stresses from other stresses using proper spatial temporal dissimilarity measurements is possible.

**Figure 3:**
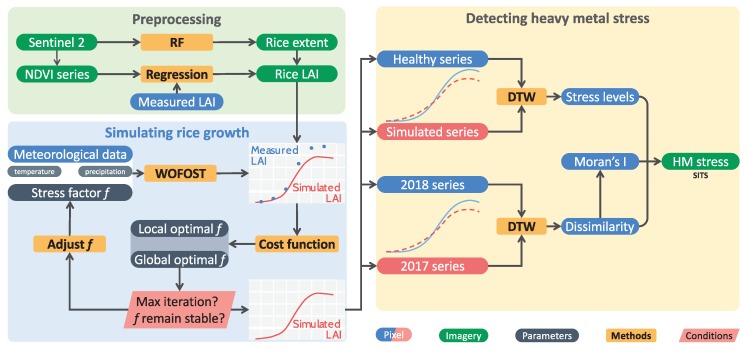
Methods to discriminate heavy metal stress in rice from multiple types of stress

Unfortunately, because of frequent cloud coverage in the study area, the temporal resolution of remote sensing images could not meet the demands of dissimilarity measurements. Thus, we used an improved WOFOST model with a stress factor to simulate the growth of rice. The overall workflow is shown in Figure 3.

### 3.1. Mapping Rice Fields

We choose random forest as the classifier to extract rice fields in Zhuzhou. A cloud-free Sentinel 2 image from September 15, 2017 was selected to map rice fields in this study. Samples were chosen on the basis of field surveys and Google Satellite imagery. A total of 85,474 pixels were selected and divided into training (80%) and testing (20%) datasets. The random forest algorithm is implemented in dzetsaka plugin by Mathieu Fauvel and Nicolas Karasiak in QGIS software for remote sensing classification [32].

Rice objects smaller than four pixels under 4-connectivity were removed and filled with the class values of the surrounding pixels after classification to assess the spatial patterns of heavy metal stress accurately.

### 3.2. Simulation of Rice LAI Dynamics

#### 3.2.1. Incorporation of Sentinel 2 Images

We selected LAI as the rice physiological parameter to assess heavy metal stress levels because leaves and other aboveground organs are prone to heavy metal stress [23]. A stress factor *f* was introduced into the WOFOST model to simulate rice LAI series dynamically under different conditions. This factor *f* was determined by Particle Swarm Optimization (PSO), which finds the optimal solution iteratively by minimizing the cost function (*C*) and stops when the maximum iteration or cost threshold is reached [33]. *C* measures the difference between the satellite-derived and simulated LAI series and is defined by Equation (1)

(1) $$\begin{array}{c} C=\sqrt{\frac{1}{N}\overset{N}{\underset{i=1}{\sum }}{\left({\mathrm{LAI}}_{m,i}-{\mathrm{LAI}}_{s,i}\right)}^{2}} \end{array}$$

(2) $$\begin{array}{c} {\mathrm{LAI}}_{m}=0.361\times \exp\left(3.69\times {\mathrm{NDVI}}_{m}\right), \end{array}$$

where ${\mathrm{LAI}}_{m,i}$ is the *i*th measurement of the LAI and derived from satellite NDVIs using the formula previously modeled in Equation (2) [34] by using field data, ${\mathrm{LAI}}_{s,i}$ is the *i*th WOFOST simulation of LAI, and *N* is the number of measurements.

#### 3.2.2. WOFOST Model

WOFOST is a physical model that dynamically simulates the daily growth of crops under different stress levels in a year.

In this study, daily sunshine hours were transformed into solar radiation power prior to the integration of the meteorological data into the WOFOST model. The relevant formulas are listed in Equations (3)–(8).

(3) $$\begin{array}{cc} D_{r} & =1+0.033\times \cos(0.0172\times \mathrm{DOY}) \end{array}$$

(4) $$\begin{array}{cc} \delta & =0.4209\times \sin(0.0172\times \mathrm{DOY}-1.39) \end{array}$$

(5) $$\begin{array}{cc} \omega_{s} & =\arccos(-\tan\varphi \cdot \tan\delta ) \end{array}$$

(6) $$\begin{array}{cc} R_{a} & =37.6\times D_{r}\left(\omega_{s}\cdot \sin\varphi \sin\delta +\cos\varphi \cos\delta \sin\omega_{s}\right) \end{array}$$

(7) $$\begin{array}{cc} N & =\frac{24}{\pi }\omega_{s} \end{array}$$

(8) $$\begin{array}{cc} R_{s} & =\left(a_{s}+b_{s}\frac{n}{N}\right)\cdot R_{a}, \end{array}$$

where DOY is the number of days since Jan 1 of a year, $D_{r}$ is the distance between the sun and Earth, δ is the declination of the Sun when Sentinel 2 images were captured, and φ is the latitude of the rice pixel (in decimal degrees). $\omega_{s}$ is the solar hour angle when Sentinel 2 satellite passes the study area, $R_{a}$ is the irradiation at the top of the aeropause, *N* is the number of daily potential sunshine hours, *n* is the actual number of sunshine hours. $a_{s}$ and $b_{s}$ are empirical constants for temperate climate zones and take the values $a_{s}=0.18$ and $b_{s}=0.55$, $R_{s}$ is the solar radiation energy.

The *f* determined by PSO is then integrated into the WOFOST model because heavy metals in soil and water may influence photosynthesis in rice. The daily total gross assimilation under stress level (${\mathrm{CVF}}_{f}$) can be defined as follows:

(9) $${\mathrm{CVF}}_{f}=f\times \mathrm{CVF},$$

where CVF is the daily total gross assimilation under the potential growth level and *f* is the stress factor, which ranges from 0 (stressed) to 1 (healthy).

To reduce the redundancy of time series and accelerate calculation, we simulated the LAI series at 5-day intervals from DOYs 160 to 255 in 2017 and 2018. This period represents the major growth season of rice in Zhuzhou area.

### 3.3. Distinguishing Heavy Metal Stress in Rice

#### 3.3.1. Conceptual Model to Distinguish Heavy Metal Stress

**Figure 4:**
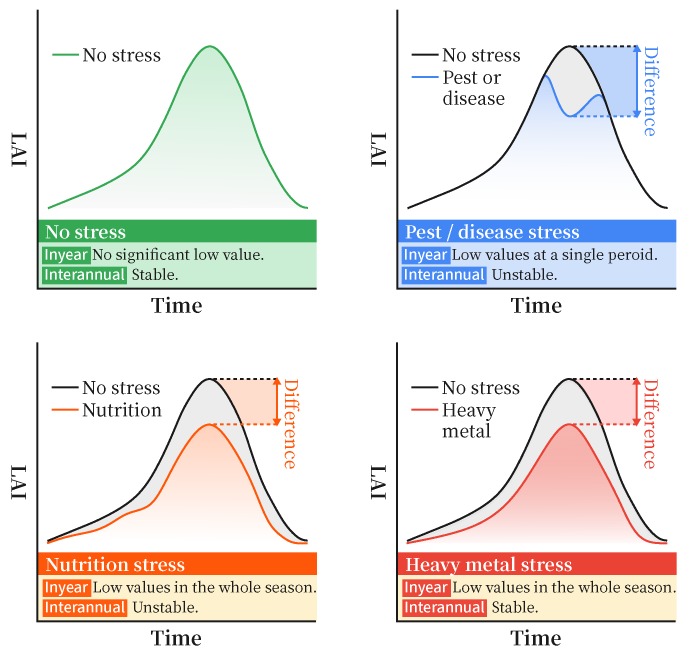
Conceptual rice series under different stress types.

The temporal features of rice LAI series under heavy metal stress are different from other stressors. Figure 4 shows the conceptual series under four major types of stress in our study area, namely pest, disease, nutrition and heavy metal stress. Temporal characteristics can be classified into two categories.

1. **In-year characteristics:** The duration of in-year signals vary when rice pixels are under different stress types. Signals of pest or disease only exist at a single period of a growing season, while signals of nutrition stress and heavy metal stress last longer and exist in the whole growing season.
2. **Inter-annual characteristics:** The variability of inter-annual signals also vary if rice pixels are under these stressors. Although nutrition stress and heavy metal stress show similar signals in a grwoing season, the variability of heavy metal stress signals are more stable than those of nutrition stress.

According to these differences in temporal characteristics, heavy metal stress can be discriminated using inter-annual stability measurements. A rice pixel is likely under heavy metal stress if its stress signals are stable in adjacent years.

#### 3.3.2. DTW

DTW is a alignment-based measurement of similarity between two time series; its goal is to find the optimal alignment between series with a minimum cost [35]. Let *Reference Series* ($A\{a_{1},a_{2},a_{3},\cdots ,a_{m}\}$) and *Query Series* ($B\{b_{1},b_{2},b_{3},\cdots ,b_{n}\}$) be two time series of lengths *m* and *n*, respectively.

**Figure 5:**
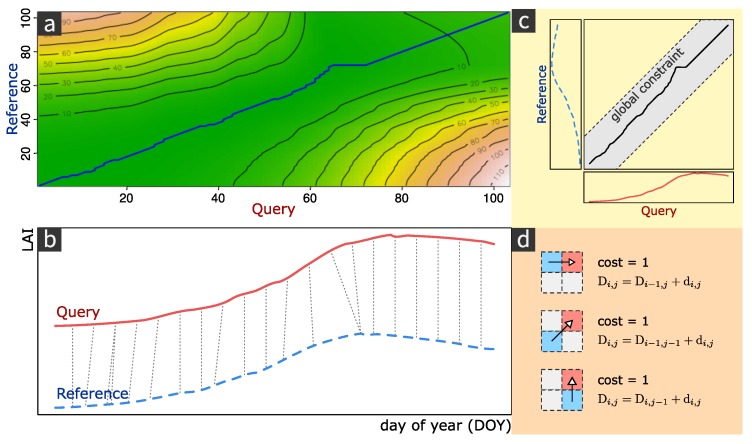
Dynamic time warping (DTW) algorithm and its constraints. (**a**) Density map of DTW warping costs. Warping costs increase from green to yellow. The blue line illustrates the warping path with the minimum cost. (**b**) DTW matches. The plot illustrates how points are related between the query series and reference series. (**c**) Global warping constraints. The search scope of the warping path is limited to a fixed distance from the main diagonal. (**d**) Local warping constraints and costs. The plot illustrates all possible choices of a warping step and the corresponding costs.

Here we define $D_{i,j}$ as the DTW distance between A(1:i) and B(1:j) and the corresponding warping path $\left(p_{i},q_{j}\right)$, illustrated in Figure 5a, is from (1,1) to (i,j). The matching nodes is illustrated in Figure 5b. Then, the DTW distance can be calculated by using Equation (10)

(10) $$D_{i,j}=\delta \left(a_{i},b_{j}\right)+\min\left\{\begin{array}{c} D(i-1,j)\\ D(i-1,j-1)\\ D(i,j-1) \end{array},\right.$$

where $\delta (a_{i},b_{j})$ is the distance measurement between node $a_{i}$ and $b_{j}$, $D_{i,j}$ is the summed distance from (1,1) to (i,j). When i=m and j=n, recursion stops and D(m,n) is the final DTW distance between series *A* and *B*.

Some constraints to the calculation of DTW distance [36] were implemented to keep the warping path continuous and accelerate calculations given that the time complexity of classic DTW is O(MN). Let node (i,j) denote points on the warping path. The constraints of the DTW algorithm can be written as follows:

- **Boundary limits**: The warping path should start from (1,1) and end in (m,n).
  (11) $$\left(p_{1},q_{1}\right)=\left(1,1\right),\left(p_{k},q_{k}\right)=\left(m,n\right).$$
- **Global constraint**: The “Sakoe Chiba Band”, illustrated in Figure 5c, was used as the global constraint to limit the warping scope to *r* samples around the main diagonal $y=\frac{n}{m}x$ [37]. Each point $\left(p_{i},q_{j}\right)$ of the warping path (p,q) should meet the following constraint:
  (12) $$\left|q_{j}-\frac{n}{m}p_{i}\right|\leq r.$$
- **Local constraints**: Local constraints are illustrated in Figure 5d. Given a node $\left(p_{i},q_{j}\right)$ from the warping path, the subsequent node should be chosen from (i+1,j), (i+1,j+1) or (i,j+1) such that the warping path is continuous and monotonically nondecreasing [38,39].

In this study, DTW was chosen for temporal dissimilarity and rice stress measurements. Temporal dissimilarity was measured for each LAI series of the same pixel between adjacent years, while stress level was derived from the DTW distance between the current and sample LAI series.

#### 3.3.3. Determination of Stress Levels in Rice

Stress levels were measured by using DTW distances from sample pixel series. To accurately detect rice stress levels, we simulated a sample series under no stress with the WOFOST model for each year. Here, the DTW distance between the LAI series of the rice pixel series (${\mathrm{LAI}}_{i}$) and the sample pixel series (${\mathrm{LAI}}_{sample}$) in the same year was defined as the stress level of the pixel ($S_{i}$) for every rice pixel (i) of a raster series. Stress levels were calculated using Equation (13) and then normalized using Equation (14)

(13) $$\begin{array}{c} S_{i}=\mathrm{dtw}\left({\mathrm{LAI}}_{sample},{\mathrm{LAI}}_{i}\right) \end{array}$$

(14) $$\begin{array}{c} S_{i,norm}=\frac{S_{i}-S_{\min}}{S_{\max}-s_{\min}}, \end{array}$$

where $S_{i,norm}$ is the normalized stress level of pixel *i*, $S_{i}$ is the original stress level in Equation (13), and $S_{\min}$ and $S_{\max}$ are the maximum and minimum stress levels, respectively. The DTW distance is positively related to the stress level, that is, a greater DTW distance from the sample series indicates that the pixel is weaker in health, and that the rice pixel is under higher stress. In this work, we applied the mean value of stress levels in 2017 and 2018 as the final stress level.

#### 3.3.4. Measurement of Temporal Dissimilarity between Rice LAI Series

In this study, temporal dissimilarity was regarded as the interannual DTW distance between the two time series of the same pixel in adjacent years (years 2017 and 2018 in this case). The temporal dissimilarity for pixel *i* was calculated by using Equation (15)

(15) $$TD_{i}=\mathrm{dtw}\left(LAI_{i,2017},LAI_{i,2018}\right),$$

where $TD_{i}$ is the temporal dissimilarity of pixel *i* and $LAI_{i,2017}$ and $LAI_{i,2018}$ are the LAI series of pixel *i* in 2017 and 2018, respectively. A small DTW distance of the same pixel indicates similar rice growth status in adjacent years.

**Figure 6:**
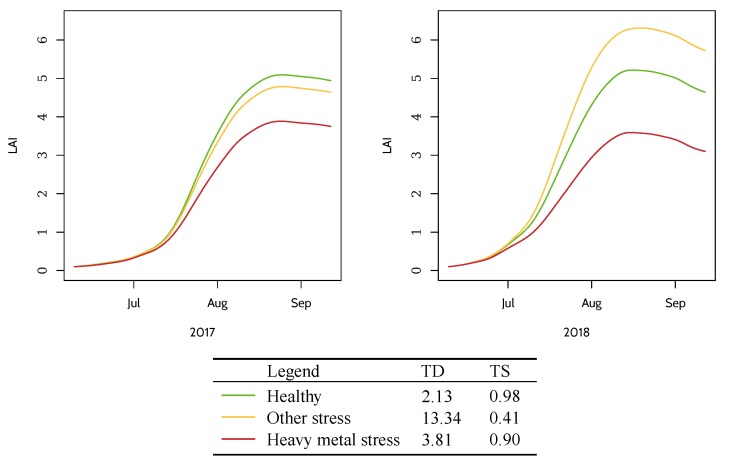
Temporal profiles of LAI series under healthy conditions, heavy metal stress and other stress conditions.

Temporal stability, defined as the normalized negative temporal dissimilarity, was transformed from temporal dissimilarity given that temporal dissimilarity is negatively correlated with temporal stability. A high temporal dissimilarity measured by DTW distance indicates low temporal stability. It is calculated using Equation (16)

(16) $$TS_{i}=1-\frac{TD_{i}-TD_{\min}}{TD_{\max}-TD_{\min}},$$

where $TS_{i}$ is the normalized temporal stability of pixel *i*, $TD_{i}$ is the original temporal dissimilarity in Equation (15), and $TD_{\min}$ and $TD_{\max}$ are minimum and maximum temporal dissimilarity, respectively. TS ranges from 0 to 1, and a high TS value indicates the rice pixel is stable and a low TS value indicates the rice pixel is unstable. As shown in Figure 6, the LAI series of rice pixels under heavy metal stress have stable and low values between 2017 and 2018 and have lower TD value (and higher TS value) than those observed in other conditions. By comparison, the LAI series under other types of stress are unstable, with a low value in 2017 and a high value in 2018, and have considerably greater TD value (and lower TS value) between adjacent years. Even though LAI series of other stress increase from year 2017 to 2018, the rice pixel is still under other stress because the stress signal lasts only for a single growing season in 2017. Heavy metal stress signals should be consistent in adjacent years. The LAI series of healthy rice pixels are stable and have high LAI values in 2017 and 2018.

TS values are expected to filter out pest, disease and nutrition stress. A high value indicates that the pixel is stable in years 2017 and 2018. But TS values alone is not sufficient because it cannot tell the difference between healthy series or heavy metal stressed series. A rice pixel is under heavy metal stress only when high TS values and low LAI values are detected. Therefore, heavy metal stress levels can only be detected with the combination of stress levels and temporal stability.

#### 3.3.5. Measurement of the Spatial Variation of Temporal Dissimilarity

Local Moran’s I, a commonly used local indicator of spatial autocorrelation, can find spatial patterns such as high-high clusters. By comparing central pixel *i* and the statistics of its neighbors *j*, the value of the central pixel can be determined to be lower or higher than that of a random distribution in space. A positive value indicates a spatial cluster and a low value indicates a spatial dispersion. In this case, a spatial dispersion indicates that the value is unstable across space, and the rice in that pixel would probably be affected by abrupt stress.

In this study, we measured the spatial patterns of DTW distances in the previous step by using local Moran’s I. The Moran’s I at pixel *i* can be calculated using Equations (17) and (18)

(17) $$\begin{array}{c} I=\frac{n}{S_{0}}\frac{\sum_{i=1}^{n}\sum_{j=1}^{n}w_{ij}\left(x_{i}-\bar{x}\left(x_{j}-\bar{x}\right)\right)}{\sum_{i=1}^{n}{\left(x_{i}-\bar{x}\right)}^{2}} \end{array}$$

(18) $$\begin{array}{c} S_{0}=\overset{n}{\underset{i=1}{\sum }}\overset{n}{\underset{j=1}{\sum }}w_{ij}, \end{array}$$

where $x_{i}$ and $x_{j}$ are temporal dissimilarities of central pixel *i* and neighbor *j*, respectively, and $w_{ij}$ is the spatial weight between *i* and *j*. $\bar{x}$ is the mean value of all rice pixels. $S_{0}$ is the sum of all spatial weights between central pixel *i* and its neighbors *j*. In this work, we used a 3×3 matrix as the neighborhood of the central pixel, and only rice pixels in the neighborhood were used for calculation.

An *I* that is significantly greater than 0, indicates the presence of a spatial cluster around pixel *i* because it is positively correlated with its neighbors. If *I* is significantly less than 0, the central pixel *i* is negatively correlated with its surrounding values. This negative correlation indicates that the central pixel may as well be in an unstable state in space. Therefore, the local Moran’s I of a rice pixel would be high if it is affected by heavy metal stress.

The local Moran’s I of pixel *i* was then normalized by using equation:

(19) $$I_{i,norm}=\frac{I_{i}-I_{\min}}{I_{\max}-I_{\min}},$$

where $I_{i,norm}$ is the normalized local Moran’s I, $I_{i}$ is the original Moran’s I of pixel *i* and $I_{\min}$ and $I_{\max}$ are the minimum and maximum local Moran’s I, respectively.

### 3.4. Construction of SIST for Assessing Heavy Metal Stress Levels

To distinguish heavy metal stress from other stresses, we considered stress levels, the temporal dissimilarity measured by DTW and local Moran’s I and developed a stress index based on spatial and temporal characteristics (SIST). SIST was calculated by using normalized stress levels ($S_{norm}$), temporal stability ($TS_{norm}$) and local Moran’s I ($I_{norm}$):

(20) $$SIST=\sqrt[{3}]{S_{norm}\times TS_{norm}\times I_{norm}}.$$

Given that SIST takes normalized factors into consideration, it should range from 0 to 1. A high SIST indicates that rice in a specific pixel is highly likely under strong heavy metal stress; a low value means the pixel is likely under weak heavy metal stress.

## 4. Results

### 4.1. Spatial Distribution of Rice Fields

The overall accuracy of the random forest classifier is 96.99%, and the Cohen’s kappa coefficient is 0.9586. The producer’s and user’s accuracy of rice are higher than 94%. The confusion matrix of the classification is shown in Table 1.

**Figure 7:**
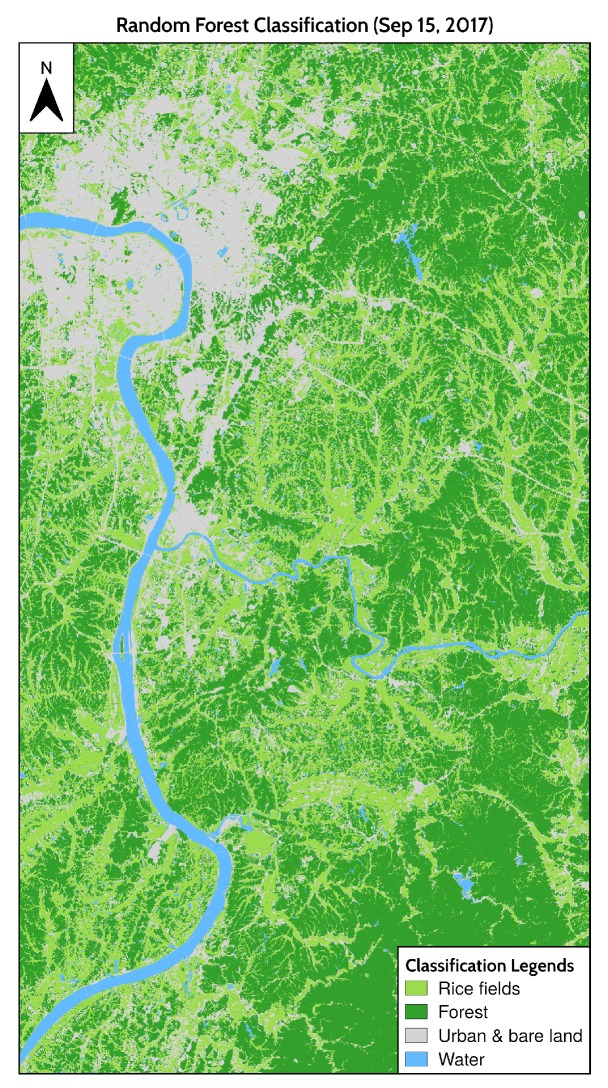
Spatial distribution of rice fields.

After classification, rice objects less than 4 pixels under 4-connectivity were removed from the rice classification results and filled with the class value of the nearest neighbors. The final classification result is shown in Figure 7.

### 4.2. Spatial Distribution of Temporal Variability and Stress Measurements

**Figure 8:**
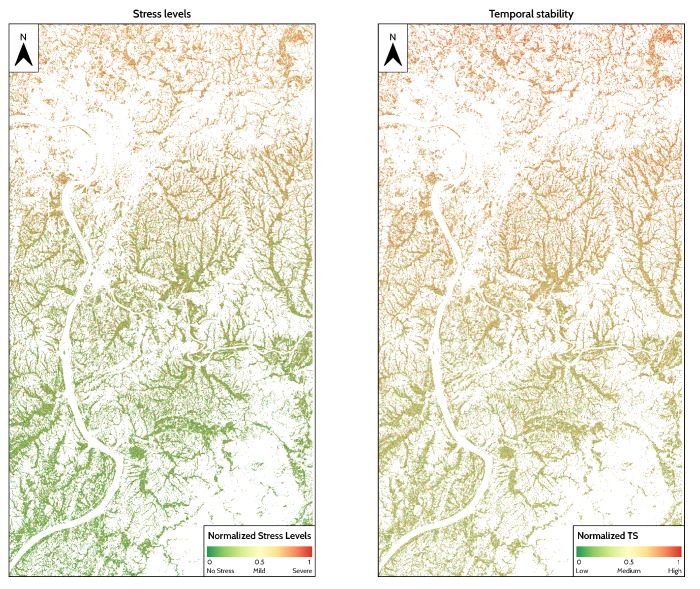
Spatial distribution of normalized stress levels and temporal stability measured by DTW. (**Left**): Normalized stress levels in 2018, ranging from 0 to 1. A high value indicates the pixel is under high stress level. (**Right**): Normalized temporal stability from 2017 to 2018 ranging from 0 to 1. A high value indicates that the pixel is stable, whereas a low value indicates that the rice pixel is unstable.

Figure 8 shows the spatial distribution of stress levels and temporal stability measured by using DTW. For temporal stability, a high DTW distance indicates that rice in a specific pixel is unstable. For stress levels, a pixel with a high DTW distance could be regarded as a high stress level, which means the rice is under stress.

Stress levels, shown in Figure 8 (Left), decreases as the distance from cities increases. In the north, rice fields tend to be under high stress levels, whereas, in the south, rice is healthy. However, temporal stability from 2017 to 2018, shown in Figure 8 (Right), shows that rice fields close to the Changsha-Zhuzhou-Xiangtan city clusters in northern areas tend to be more stable than those in the south, which is distant from industrial parks and cities.

### 4.3. Spatial Distribution of Heavy Metal Stress Levels in Rice

**Figure 9:**
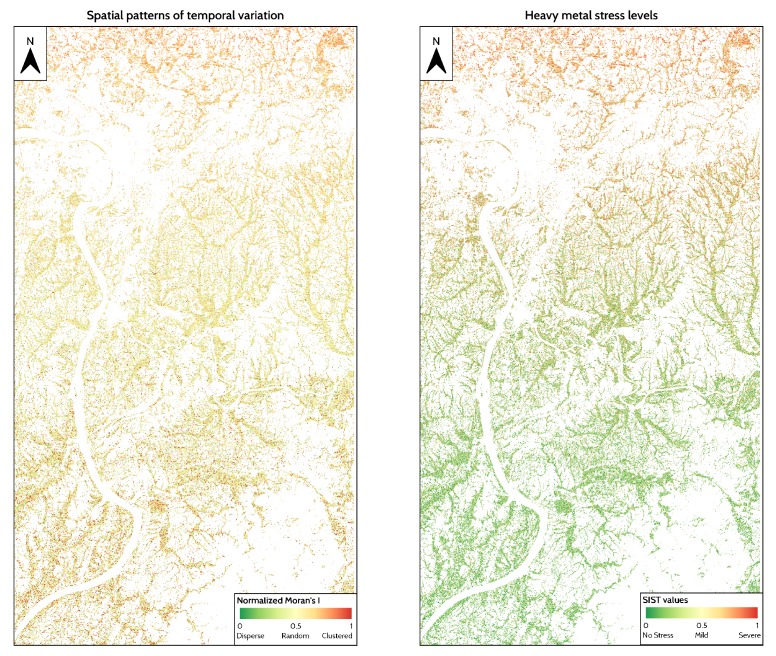
Spatial distribution of Moran’s I of temporal stability and stress index based on spatial and temporal characteristics (SIST). (**Left**): Normalized Moran’s I of temporal stability, ranging from 0 to 1. Values close to 1 indicate that the pixel tends to be clustered, 0.5 indicates perfect randomness, and 0 indicates complete dispersion. (**Right**): Spatial distribution of normalized SIST ranging from 0 to 1. A lower value of SIST (greener) indicates the rice pixel is less likely to be under heavy metal stress

Figure 9 shows high heavy metal stress levels in the industrial areas and lower levels in rural areas. This trend is similar to the trend of general stress levels. High-high clusters are defined as places where local Moran’s I is greater than 0 and have high TS values, low-low clusters are defined as places where local Moran’s I is greater than 0 and have low TS values.

Heavy metal-stressed rice pixels and healthy pixels have high Moran’s I value, indicating that under nonstress or heavy metal stress, rice growth shows a spatial pattern of clustering. The only difference between the two spatial patterns is that healthy rice pixels are in low-low TS clusters, whereas heavy metal stressed ones are in high-high TS clusters. In the center of the study area near Zhuzhou County, where heavy metal stress is at a moderate level, Moran’s I is close to 0, which implies the presence of other types of abrupt stressors, such as pests and diseases in that area.

### 4.4. Spatial Patterns of Rice under Different Stress Types

As illustrated in Figure 9, the spatial distribution of SIST, temporal stability, and stress levels shows similar trends with a high degree of heavy metal stress in the northern areas and a low degree in the south. The Moran’s I of temporal stability is close to 0 in the central region, where heavy metal stress is at a moderate level, and shows no significant spatial clustering patterns. Heavy metal-stressed areas and healthy fields show a high Moran’s I, indicating that, under both conditions, temporal stability shows a spatial pattern of clustering.

## 5. Discussion

### 5.1. How DTW Works in Temporal Dissimilarity Measurement

**Figure 10:**
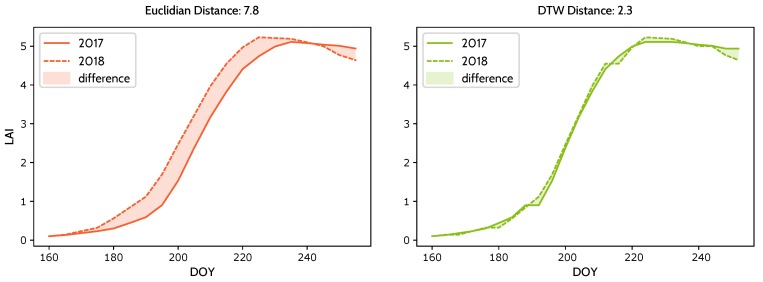
Two healthy and stable LAI series which may be considered to be under stress if we use Euclidian distance. X axis denotes day of year in 2017 and 2018, y axis denotes the LAI value of the pixel. (**Left**): Original series and difference measured by Euclidian distance. (**Right**): Warped series using DTW and difference measured by DTW.

In this article, we choose DTW over Euclidian distance as the measurement of temporal dissimilarity. The reason is that DTW can eliminate the unwanted distance caused by different timings of rice phenology stages. Take the two series in Figure 10 for example. The two series are LAI series from the same pixel, and the major difference between them is the timing, not amplitude. Timings of rice phenology stages vary from year to year, so this different timing can result in unwanted increase in distance measurement. The distance between the two series is 7.8 if we use Euclidian distance, but for DTW distance, the distance is 2.3, which is much smaller.

### 5.2. Correlation between SIST and Heavy Metal Concentration

Soil Cd concentrations (mg/kg) in Section 2.2.1 is used as reference data to assess the accuracy of SIST in detecting heavy metal stress levels. Pearson correlation coefficient (*r*) [40] is chosen as the accuracy measurement. Given two variables *x* and *y*, *r* is calculated using the following formula:

(21) $$r=\frac{\sum_{i=1}^{n}\left(x_{i}-\bar{x}\right)\left(y_{i}-\bar{y}\right)}{\sqrt{\sum_{i=1}^{n}{\left(x_{i}-\bar{x}\right)}^{2}}\sqrt{\sum_{i=1}^{n}{\left(y_{i}-\bar{y}\right)}^{2}}},$$

where *n* is sample size, $x_{i}$ and $y_{i}$ are SIST values and Cd concentrations (mg/kg), respectively. $\bar{x}$ and $\bar{y}$ are mean values of SIST and Cd concentrations, respectively.

**Figure 11:**
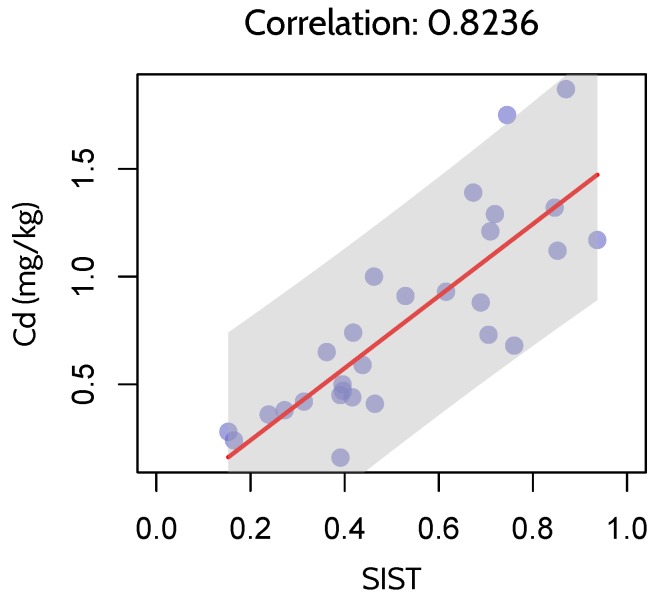
Correlation between SIST and Cd concentrations.

As is shown in Figure 11, SIST is positively correlated with Cd concrations, with a *r* of 0.8236. This high correlation between SIST and Cd concentrations demonstrates that SIST is correlated with heavy metal stress levels in rice.

### 5.3. Relationship between Heavy Metal Stress Levels and Industrial Activities

**Figure 12:**
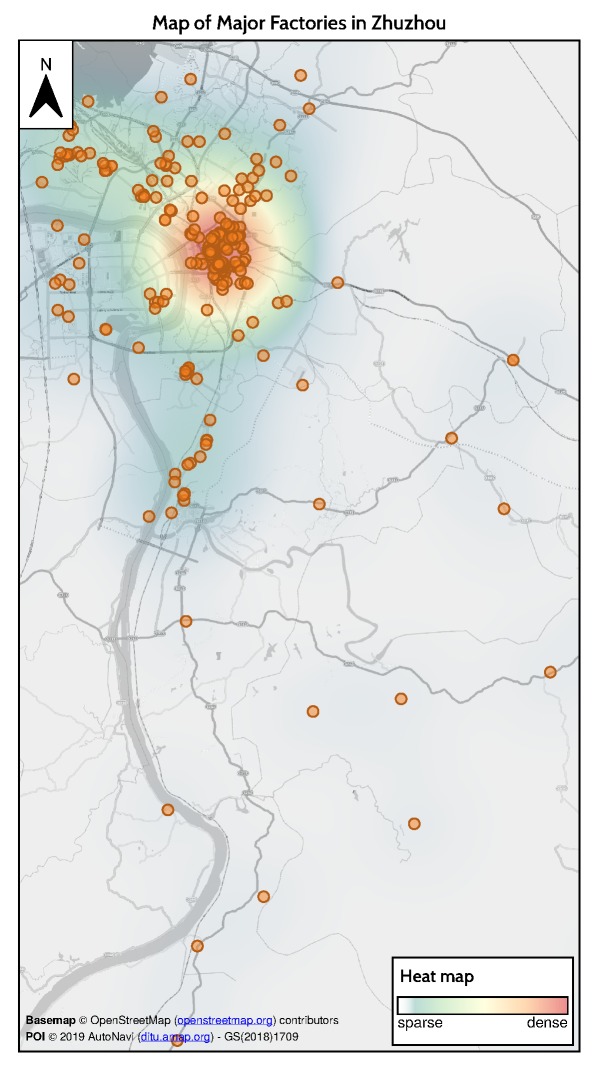
Spatial distribution and heat map of major factories in Zhuzhou.

To identify the relationship between heavy metal stress in rice and industrial activities, we searched for factories that may cause heavy metal contamination by using AutoNavi, one of the largest online map services in China. Among various types of factories, those associated with metallurgy and machine manufacturing contribute greatly to heavy metal pollution, pose threats to the surrounding soil, and negatively affect rice growth. The spatial distribution of these factories are shown in Figure 12.

From the spatial distribution of factories in Zhuzhou, we can find that most factories are located north of Zhuzhou City, where SIST values are high. SIST values near Zhuzhou County in the center of the study area, where factories are scarce, are much lower than those in northern areas but still noticeably higher than those in southern rural areas. The spatial distribution of high-pollution factories is mostly consistent with that of SIST.

### 5.4. Advantages and Disadvantages of SIST

SIST can distinguish long-term stable stress signals such as heavy metals in rice, from multiple types of stress. Given that the impact of heavy metal stress on rice is stable across space and time and exhibits similar stress signals between adjacent years and in surrounding fields, SIST can be used to distinguish heavy metal stress levels by taking stress levels, temporal variation, and spatial patterns of a rice pixel into consideration. In this way, the signals of abrupt stress (pest and diseases), which may be unstable in one or more life cycles, are eliminated and only long-term stress signals retained. DTW is used in temporal dissimilarity measurement to eliminate the temporal scaling and shifting effects of different rice phenology stages, thereby increasing the accuracy of temporal dissimilarity.

However, SIST shows several limitations in the measurement of heavy metal stress levels. First, SIST is based on the assumption that a pixel should not be under a composite of heavy metal stress and other stresses at the same time. It cannot distinguish heavy metal stress if the rice pixel is under abrupt stress (pest and disease) as well. Second, the calculation of SIST requires a dense and long time series. This requirement necessitates the use of at least a 2-year-long time series with a suitable temporal interval in a growing season.

### 5.5. Limitations of This Study

There are some limitations of this study although heavy metal stress signals are captured by SIST effectively. First, we assumed that the relationship between LAI and NDVI remain unchanged in 2017 and 2018, which might introduce some uncertainty to heavy metal stress detection. Second, temporal stability was measured based on two-year-long time series. Although pest, disease and nutrition stress vary from year to year, two years’ observations may be insufficient to distinguish heavy metal stress in rice, which might also introduce some errors to temporal stability measurements. We will take longer time series for analysis in our future research. Finally, Cd concentration in soil is an indirect reflection of heavy metal stress levels in rice. The relationship between Cd concentrations and heavy metal stress levels is not modeled here, which might bring some uncertainties to the study as well.

## 6. Conclusions

In this study, we used DTW to measure stress levels, temporal stability, and its spatial patterns of rice LAI series. By comparing series of the same pixel in adjacent years, the temporal dissimilarity can be measured, and temporal stability can then be calculated. Heavy metal stress can be distinguished from other types of stress using these spatio-temporal characteristics of rice LAI series. We also introduced a stress index based on these features (SIST) to assess heavy metal stress levels in rice. The results demonstrate that

1. Heavy metal stress can be discriminated by extracting temporal characteristics of rice LAI series because unlike signals of other types of stress, heavy metal stress signals are stable during the whole growing season, and show similar temporal profiles in different years.
2. Spatial patterns of rice temporal features, measured by local Moran’s I, can help to discriminate heavy metal stress because heavy metal stress tend to be clustered while abrupt stress tend to be random in space.
3. SIST, a spatio-temporal index based on time series of leaf area index, can detect heavy metal stress by taking both temporal and spatial features into consideration. The high correlation between SIST and heavy metal concentrations demonstrates that SIST is capable of this task.
4. The difference between temporal profiles of rice LAI series under heavy metal stress and other stress types could be accurately discriminated using DTW because it can eliminate the influence of the different timings of phenological stages on rice growth, which is quite common for crops in different years or locations. DTW might be suitable for other time series based applications like forest disturbance detection.

## Figures and Tables

**Table 1:** Confusion matrix of land-use types using random forest.

	Rice	Forest	Urban	Water	User’s Accuracy
Rice	4089	136	73	13	95.85%
Forest	72	3008	31	3	96.60%
Urban	79	65	6272	26	97.36%
Water	6	2	8	3213	99.50%
Producer’s accuracy	96.30%	93.68%	98.25%	98.71%	96.99%

## References

[1] Du F., Yang Z., Liu P., Wang L. (2018). Accumulation, translocation, and assessment of heavy metals in the soil-rice systems near a mine-impacted region. Environ. Sci. Pollut. Res..

[2] Cao H., Chen J., Zhang J., Zhang H., Qiao L., Men Y. (2010). Heavy metals in rice and garden vegetables and their potential health risks to inhabitants in the vicinity of an industrial zone in Jiangsu, China. J. Environ. Sci..

[3] Liu H., Zhang J., Christie P., Zhang F. (2008). Influence of iron plaque on uptake and accumulation of Cd by rice (Oryza sativa L.) seedlings grown in soil. Sci. Total. Environ..

[4] Duruibe J.O., Ogwuegbu M., Egwurugwu J. (2007). Heavy metal pollution and human biotoxic effects. Int. J. Phys. Sci..

[5] Fu J., Zhou Q., Liu J., Liu W., Wang T., Zhang Q., Jiang G. (2008). High levels of heavy metals in rice (Oryzasativa L.) from a typical E-waste recycling area in southeast China and its potential risk to human health. Chemosphere.

[6] Wang F., Gao J., Zha Y. (2018). Hyperspectral sensing of heavy metals in soil and vegetation: Feasibility and challenges. ISPRS J. Photogramm. Remote. Sens..

[7] Zhang Z., Liu M., Liu X., Zhou G. (2018). A New Vegetation Index Based on Multitemporal Sentinel-2 Images for Discriminating Heavy Metal Stress Levels in Rice. Sensors.

[8] Zhang B., Liu X., Liu M., Wang D. (2017). Thermal infrared imaging of the variability of canopy-air temperature difference distribution for heavy metal stress levels discrimination in rice. J. Appl. Remote. Sens..

[9] Liu F., Liu X., Zhao L., Ding C., Jiang J., Wu L. (2015). The Dynamic Assessment Model for Monitoring Cadmium Stress Levels in Rice Based on the Assimilation of Remote Sensing and the WOFOST Model. IEEE J. Sel. Top. Appl. Earth Obs. Remote. Sens..

[10] Tian L., Liu X., Zhang B., Liu M., Wu L. (2017). Extraction of Rice Heavy Metal Stress Signal Features Based on Long Time Series Leaf Area Index Data Using Ensemble Empirical Mode Decomposition. Int. J. Environ. Res. Public Health.

[11] Liu M., Liu X., Li M., Fang M., Chi W. (2010). Neural-network model for estimating leaf chlorophyll concentration in rice under stress from heavy metals using four spectral indices. Biosyst. Eng..

[12] Yang C.M., Cheng C.H., Chen R.K. (2007). Changes in spectral characteristics of rice canopy infested with brown planthopper and leaffolder. Crop Sci..

[13] Liu M., Wang T., Skidmore A.K., Liu X. (2018). Heavy metal-induced stress in rice crops detected using multi-temporal Sentinel-2 satellite images. Sci. Total. Environ..

[14] Nelson A., Setiyono T., Rala A.B., Quicho E.D., Raviz J.V., Abonete P.J., Maunahan A.A., Garcia C.A., Bhatti H.Z.M., Villano L.S. (2014). Towards an Operational SAR-Based Rice Monitoring System in Asia: Examples from 13 Demonstration Sites across Asia in the RIICE Project. Remote Sens..

[15] Getis A., Ord J.K. (1995). Local Spatial Autocorrelation Statistics: Distributional Issues and an Application. Geogr. Anal..

[16] Bellman R., Kalaba R. (1959). On adaptive control processes. IRE Trans. Autom. Control..

[17] Senin P. (2008). Dynamic Time Warping Algorithm Review.

[18] Berndt D.J., Clifford J. (1994). Using Dynamic Time Warping to Find Patterns in Time Series.

[19] Belgiu M., Csillik O. (2018). Sentinel-2 cropland mapping using pixel-based and object-based time-weighted dynamic time warping analysis. Remote Sens. Environ..

[20] Maus V., Câmara G., Cartaxo R., Sanchez A., Ramos F.M., de Queiroz G.R. (2016). A Time-Weighted Dynamic Time Warping Method for Land-Use and Land-Cover Mapping. IEEE J. Sel. Top. Appl. Earth Obs. Remote Sens..

[21] Petitjean F., Inglada J., Gancarski P. (2012). Satellite Image Time Series Analysis Under Time Warping. IEEE Trans. Geosci. Remote Sens..

[22] Van Diepen C., Wolf J., van Keulen H., Rappoldt C. (1989). WOFOST: A simulation model of crop production. Soil Use Manag..

[23] Wu L., Liu X., Wang P., Zhou B., Liu M., Li X. (2013). The assimilation of spectral sensing and the WOFOST model for the dynamic simulation of cadmium accumulation in rice tissues. Int. J. Appl. Earth Obs. Geoinf..

[24] Curnel Y., de Wit A.J., Duveiller G., Defourny P. (2011). Potential performances of remotely sensed LAI assimilation in WOFOST model based on an OSS Experiment. Agric. For. Meteorol..

[25] Ma G., Huang J., Wu W., Fan J., Zou J., Wu S. (2013). Assimilation of MODIS-LAI into the WOFOST model for forecasting regional winter wheat yield. Math. Comput. Model..

[26] Boogaard H., Van Diepen C., Rotter R., Cabrera J., Van Laar H. (1998). WOFOST 7.1; User’s Guide for the WOFOST 7.1 Crop Growth Simulation Model and WOFOST Control Center 1.5.

[27] Jin M., Liu X., Wu L., Liu M. (2017). Distinguishing heavy-metal stress levels in rice using synthetic spectral index responses to physiological function variations. IEEE J. Sel. Top. Appl. Earth Obs. Remote. Sens..

[28] Anselin L. (1995). Local Indicators of Spatial Association—LISA. Geogr. Anal..

[29] Evil Transform. https://github.com/googollee/eviltransform.

[30] Gorelick N., Hancher M., Dixon M., Ilyushchenko S., Thau D., Moore R. (2017). Google Earth Engine: Planetary-scale geospatial analysis for everyone. Remote Sens. Environ..

[31] Fu J., Zhang A., Wang T., Qu G., Shao J., Yuan B., Wang Y., Jiang G. (2013). Influence of E-Waste Dismantling and Its Regulations: Temporal Trend, Spatial Distribution of Heavy Metals in Rice Grains, and Its Potential Health Risk. Environ. Sci. Technol..

[32] Dzetsaka Qgis Classification Plugin. https://github.com/nkarasiak/dzetsaka.

[33] Kennedy J., Eberhart R. Particle swarm optimization. Proceedings of the ICNN’95—International Conference on Neural Networks.

[34] Zhou G., Liu X., Zhao S., Liu M., Wu L. (2017). Estimating FAPAR of rice growth period using radiation transfer model coupled with the WOFOST model for analyzing heavy metal stress. Remote Sens..

[35] Zhang Z., Tavenard R., Bailly A., Tang X., Tang P., Corpetti T. (2017). Dynamic Time Warping under limited warping path length. Inf. Sci..

[36] Keogh E.J., Pazzani M.J. Derivative Dynamic Time Warping. Proceedings of the 2001 SIAM International Conference on Data Mining, Proceedings, Society for Industrial and Applied Mathematics.

[37] Sakoe H., Chiba S. (1978). Dynamic programming algorithm optimization for spoken word recognition. IEEE Trans. Acoust. Speech, Signal Process..

[38] Giorgino T. (2009). Computing and Visualizing Dynamic Time Warping Alignments in R: The dtw Package. J. Stat. Softw..

[39] Spooner M., Kold D., Kulahci M. (2017). Selecting local constraint for alignment of batch process data with dynamic time warping. Chemom. Intell. Lab. Syst..

[40] Lee Rodgers J., Nicewander W.A. (1988). Thirteen ways to look at the correlation coefficient. Am. Stat..

